# Outcomes from the Introduction of a Combined Urology Outpatient Clinic

**DOI:** 10.1155/2018/9738548

**Published:** 2018-11-29

**Authors:** Clíodhna Browne, Catherine M. Dowling, Patrick O'Malley, Nadeem Nusrat, Kilian Walsh, Syed Jaffry, Eamonn Rogers, Garrett C. Durkan, Frank T. D'Arcy

**Affiliations:** Department of Urology, University College Hospital Galway, Newcastle Road, Galway, Ireland

## Abstract

**Background:**

A combined urology clinic staffed by four consultants and four non‐consultant hospital doctors (NCHDs) was introduced in our institution in October 2015. This clinic is supported by a pre‐clinic radiology meeting and a synchronous urology clinical nurse specialist (CNS) clinic with protected uroflow/trial of void slots. Herein, we report on the outcomes of this clinic in comparison with the standard format of urology outpatient review.

**Methods:**

We carried out a retrospective review of clinic attendances from May to July 2016. We recorded the number of new and return attendances, which team members had reviewed the patient and patient outcomes. We also calculated the waiting times for new patients to be reviewed in the outpatient clinic.

**Results:**

The combined urology clinic reviewed an average of 12 new and 46 return patients per clinic. The standard urology clinic reviewed an average of 8 new and 23 return patients per clinic. 54% of patients were seen by a consultant in the combined urology clinic, and 20% of patients were seen by a consultant in the standard urology clinic. The rate of patient discharge for new patients was 14.8% in the combined clinic compared to 5.9% in the standard clinic. Overall patient outcomes are outlined in the table. The waiting time for review of new patients in the combined clinic was reduced by 39% from 144 days to 89 days over a one-year period.

**Conclusions:**

The introduction of a combined urology outpatient clinic with the support of pre‐clinic radiology meeting and synchronous urology CNS clinic facilitates patient discharge.

## 1. Introduction

Urology is a speciality with a large volume of outpatient work. Hospital outpatient services make up a significant proportion of urologists' working hours. Manpower and workforce crisis issues have a significant impact on outpatient services in our hospitals. The British Association of Urological Surgeons (BAUS) has produced guidelines that suggest ideal clinic numbers and outline the proportion of the working week that should be dedicated to outpatient clinics. In many urology departments, these numbers are not adhered to, due to long outpatient waiting lists and unfilled consultant posts. It has been suggested that a comprehensive combined “one-stop” clinic review, incorporating imaging and endoscopy, would facilitate discharge of patients after one clinic visit.

A combined urology clinic staffed by four consultant urologists with different subspecialist interests was introduced in our institution in October 2015. A total of eight doctors staff the combined clinic, four consultant urologists and four non‐consultant hospital doctors (NCHDs). Every second week, there were three consultants and three NCHDs in attendance. In comparison with the standard clinic, the combined clinic staffing structure is such that although an equivalent number of patients per doctor are seen in both clinics, there are more consultants present in the combined clinic compared to the standard clinic. The intention of the increased consultant to patient ratio compared to the standard clinic is to facilitate early senior decision-making and fast-track the patient journey through the outpatient system.

Clinic lists are reviewed by the consultant in advance. Patients attending for follow-up of normal results are often contacted by phone and letter as a virtual clinic review, negating the need for them to travel to the clinic. The combined clinic is supported by a pre‐clinic radiology meeting in the style of a standard multidisciplinary team meeting, where patient's imaging is reviewed by a consultant radiologist in the presence of the clinic team. This meeting lasts one hour and happens immediately before the clinic. Individual cases are discussed to plan surgical approaches or patient follow-up as required. A synchronous urology clinical nurse specialist (CNS) clinic with protected slots for uroflow measurements and trials of voiding runs in tandem with the combined urology clinic. Herein, we aim to report on the outcomes of the combined clinic in comparison with the standard format of the urology outpatient clinic that was traditionally employed in this institution.

## 2. Methods and Materials

We carried out a retrospective review of clinic attendances from a representative time period after pilot introduction of the combined clinic (May to July 2016). We recorded the number of new and return attendances, which team members had reviewed the patient and patient outcomes. We also calculated the waiting times for new patients to be reviewed in the outpatient clinic. These outcomes were compared to the clinic attendances at the standard urology clinic over the same time period.

## 3. Results

### 3.1. Referral Sources

A total of 757 patients attended the combined urology clinic over the study period. There were 183 new patients and 574 return patients seen in the combined clinic. A total of 372 patients attended the standard urology clinic over the study period. There were 118 new patients and 254 return patients in this group. The combined urology clinic reviewed an average of 12 new and 46 return patients per clinic. The standard urology clinic reviewed an average of 8 new and 23 return patients per clinic. The staffing structure of the clinics as outlined above means that the combined clinic reviewed an average of 17 patients per whole-time equivalent consultant compared to an average of 31 patients per whole-time equivalent consultant in the standard clinic.

**Figure 1:**
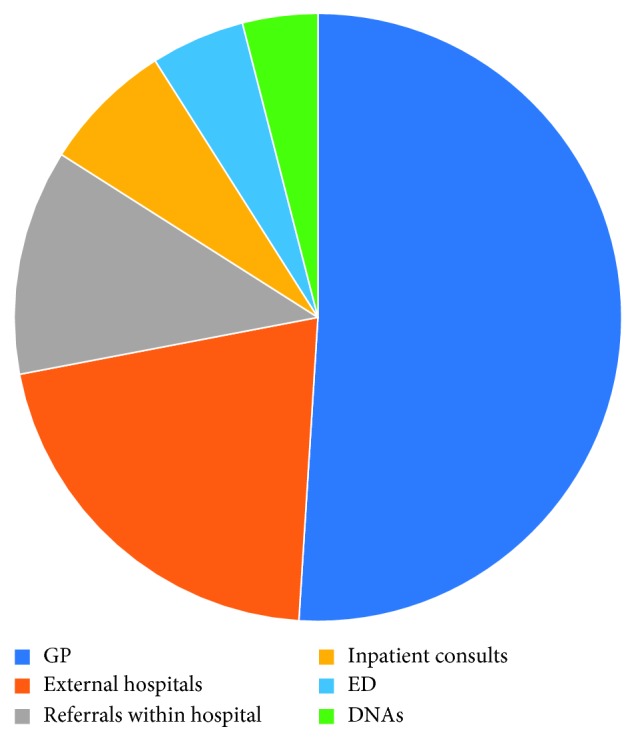
Standard urology clinic referrals.

**Figure 2:**
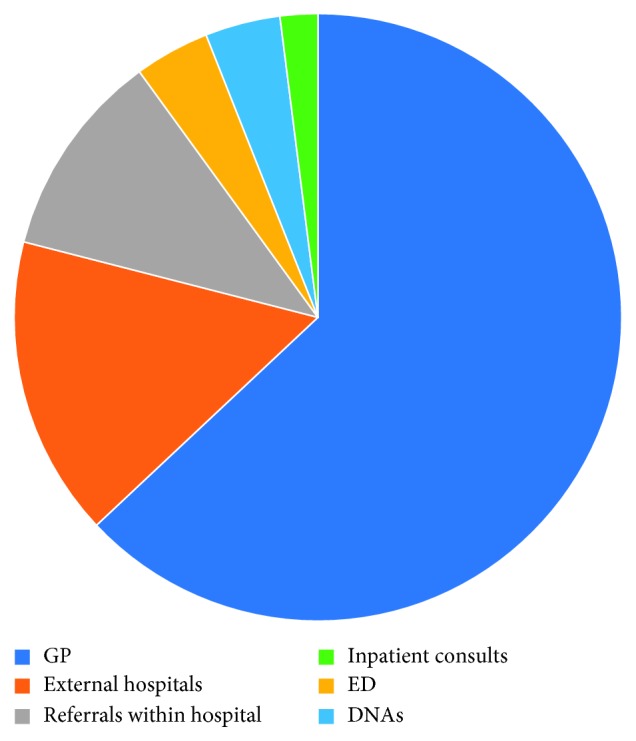
Combined urology clinic referrals.

The sources of new patient referrals to the combined urology clinic were 51% from general practitioners, 21% from other hospitals, 12% from other consultants in our hospital, 7% from inpatient consultations, 5% from the emergency department, and 4% from previous clinic nonattendances (Figure 1). The sources of new patient referrals to the standard urology clinic were 63% from general practitioners, 16% from other hospitals, 11% from other consultants in our hospital, 4% from the emergency department, 4% from previous clinic non‐attendances, and 2% from inpatient consultations (Figure 2).

### 3.2. Waiting Times

The median waiting time for new patients to be seen in the combined clinic was 53 days. The median waiting time for new patients to be seen in the standard urology clinic was 98 days. The mean waiting time for new patients was reduced by 38% during the first year of the combined urology clinic.

### 3.3. Patient Reviews

Fifty-four percent of patients were seen by a consultant in the combined urology clinic, and 20% of patients were seen by a consultant in the standard urology clinic. A consultant reviewed 56% of the new patients in the combined clinic and 21% of new patients in the standard urology clinic.

### 3.4. Patient Outcomes

Overall patient outcomes are outlined in Table 1. Of the patients attending the combined urology clinic, 51.8% were booked for a further clinic review, 21.1% were discharged, 26.8% were booked for a procedure, and 0.3% were admitted. Of the patients attending the standard urology clinic, 55.4% were booked for further clinic review, 34.1% were booked for a procedure, 10.2% were discharged, and 0.3% were admitted. The rate of patient discharge for new patients was 14.8% in the combined clinic compared to 5.9% in the standard clinic.

## 4. Discussion

Manpower and workforce crisis issues have a significant impact on outpatient services in Irish hospitals. The British Association of Urological Surgeons (BAUS) guidelines suggest ideal clinic numbers of eleven new patients and fifteen return patients per consultant per outpatient clinic [1]. In total, combined new and review patients should be limited at 12 patients per consultant per clinic. BAUS recommends allocating twenty minutes per consultation for new patients and two to fifteen minutes per consultation for review patients. Complex patients referred for specialist opinion should be allocated thirty to forty-five minutes per consultation. These numbers are rarely adhered to in practice due to restrictions on clinician time and prohibitively long waiting lists. Indeed, the clinic numbers reported in this study significantly exceed these recommended numbers.

Gilmore et al. found that only 25% of Irish urologists and 21% of UK urologists had outpatient practices that would fall within the guidelines issues by BAUS [2]. This group reported a median of two outpatient clinics per department per week, with thirteen new and twenty-six return patients. The numbers in our institution are similar to those outlined in this study. The numbers attending the combined urology clinic are higher, but this clinic is staffed by four different consultant urologists.

The ideal situation for urology outpatient services is full access to comprehensive combined “one-stop” clinic review that incorporates imaging and endoscopy. Some centres have implemented a similar strategy in the form of rapid access haematuria clinics with access to laboratory blood tests, upper tract imaging, and flexible cystoscopy. Traditionally, several clinic visits were required to diagnose one issue. Coull et al. reported on the implementation of a “one-stop” diagnostic clinic for urology patients [3]. They report an impressive 42% discharge rate back to GP care during the pilot period of this clinic. While neither clinic in this study has reached such discharge rates, the discharge rate in the combined clinic is double that of the standard clinic. This is likely as a result of the increased number of senior decision-makers in attendance at the clinic and the access to adjuncts such as the pre‐clinic radiology meeting and CNS clinic. Availability of same-day imaging and endoscopy in our combined clinic would likely further improve outpatient efficiency.

Combined clinics have been reported in the literature in a variety of different complimentary specialities, including ophthalmology, renal disease, diabetes, cardiovascular disease, orthopaedics, and rheumatology. Patient outcomes have been favourable in these studies, with reductions in the number of clinic visits required for individual patients [4–7]. These approaches have also proven to be cost effective. Weber et al. reported on a group of patients with multiple related medical issues (kidney disease, cardiovascular disease, and diabetes) who attended a combined clinic with all the relevant speciality doctors, rather than individual speciality clinics [8]. Hospitalisation, dialysis, and mortality rates were no different between patients in the combined group and patients attending the individual clinics. However, differences in the cost of clinic visits were $86,400 per year in favour of the combined clinic.

The most significant difference in the results from our clinic was the rate of patients discharged versus those booked for further investigations or procedures. This can most likely be explained by a number of factors unique to the combined clinic. The pre‐clinic X-ray conference enables a comprehensive review of imaging with a consultant radiologist in a formal multidisciplinary team setting. This informs the discussion with the patient and assists with decision-making, e.g., regarding follow-up of renal cysts or in planning operative approach in complex stone disease. The better consultant to patient ratio facilitates senior decision-making in a timely fashion, reducing the number of patients being sent for unnecessary investigations and increasing patient discharge rates. The combined clinic is staffed by four consultants with different subspecialist interests. This enables cross-referral between consultants, further facilitating senior decision-making and negating the need for return outpatient visits to see the appropriate surgeon.

## 5. Conclusions

The introduction to our department of a combined urology outpatient clinic with the support of pre‐clinic radiology meeting and synchronous urology CNS clinic has facilitated patient discharge and enabled prompt senior decision-making for our outpatients. The presence of a senior decision-maker results in more consultant-led care and facilitates patient discharge. The introduction of same-day imaging and endoscopy would likely further improve outpatient clinic efficiency.

## Figures and Tables

**Table 1:** Outcomes of clinic review.

Patient outcome	Combined clinic (%)	Standard clinic (%)
Discharged	21.1	10.2
Procedure booked	26.8	34.1
Admitted	0.3	0.3
Further clinic review	51.8	55.4

## Data Availability

The data used to support the findings of this study are available from the corresponding author upon request.
